# Supplementary material for: Tea-Derived Polyphenols Enhance Drought Resistance of Tea Plants (Camellia sinensis) by Alleviating Jasmonate–Isoleucine Pathway and Flavonoid Metabolism Flow
Source: Int J Mol Sci. 2024 Mar 29;25(7):3817. doi: 10.3390/ijms25073817 (PMC11011871; doi:10.3390/ijms25073817)
Supplement: Supplementary file 1 [file ijms-25-03817-s001.zip › Figures S1-S9.pdf]

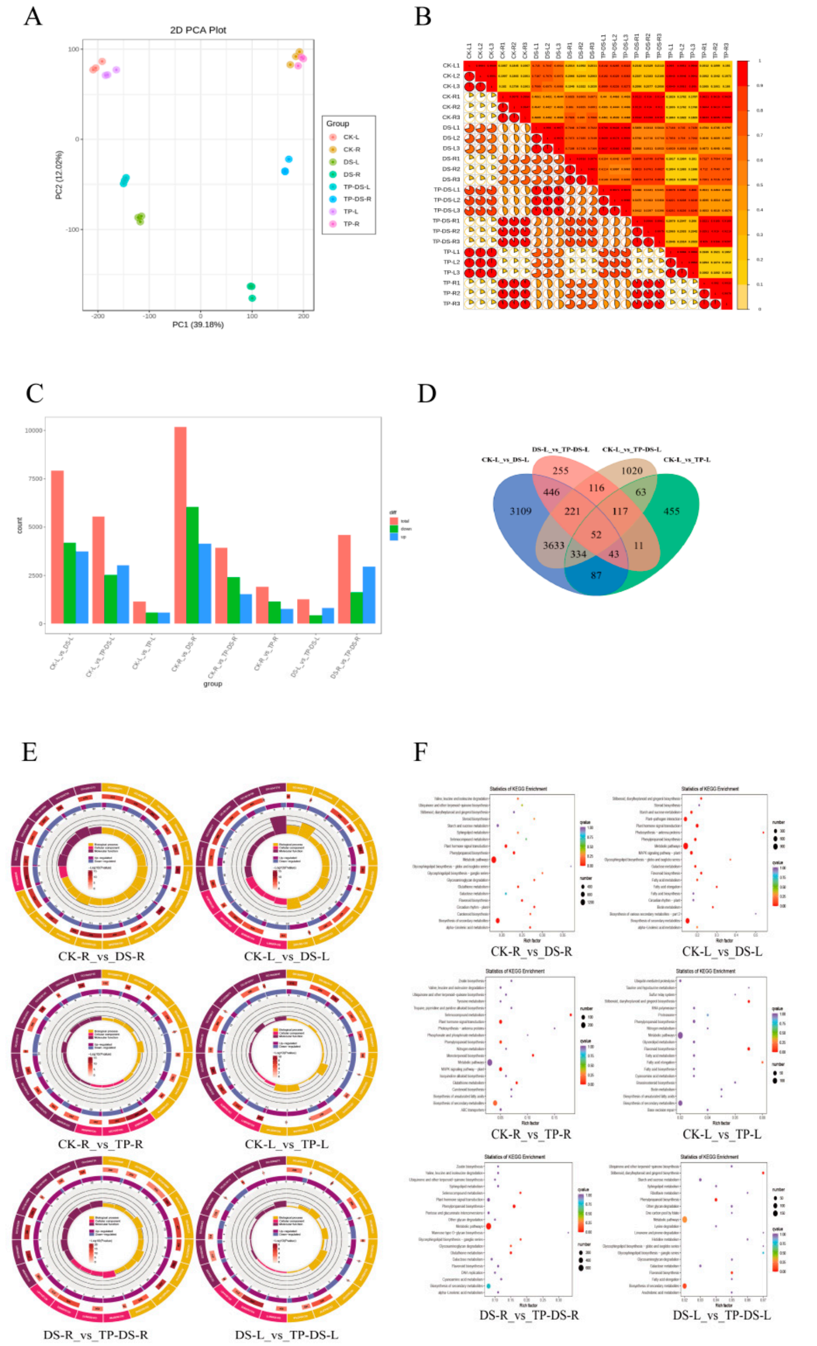

Figure S1. Transcriptome analysis of leaves and roots under different treatments. Transcriptome (**A**) PCA and (**B**) Pearson correlation analysis of leaf and root transcriptomes of tea seedlings in different treatments under drought; (**C**,**D**) Overall histograms of differentially expressed genes and Venn diagrams of leaf differential genes; (**E**,**F**) Circle diagrams of GO enrichment analysis of differentially expressed genes and KEGG-enriched scatter plots. Yellow for biological processes; red for cellular components; purple for molecular functions. The ordinate represents the KEGG pathway. The abscissa represents the Rich factor. Rich factor refers to the ratio of the enriched differential genes (Sample number) to the annotated genes (Background number) in the pathway. The greater the Rich factor, the greater the degree of enrichment. The larger the dot, the higher the number of differential genes enriched by the pathway. P-value: p-value for significance test; Corrected_P-value (qvalue): p-value after correction for multiple hypothesis test The smaller the qvalue, the redder the color of the point, indicating the more significant the enrichment. CK: control group, normal watering; TP: exogenous tea polyphenol pretreatment and normal watering; TP+DS: exogenous tea polyphenol pretreatment and drought treatment; DS: drought treatment; L: leaves; R: roots.

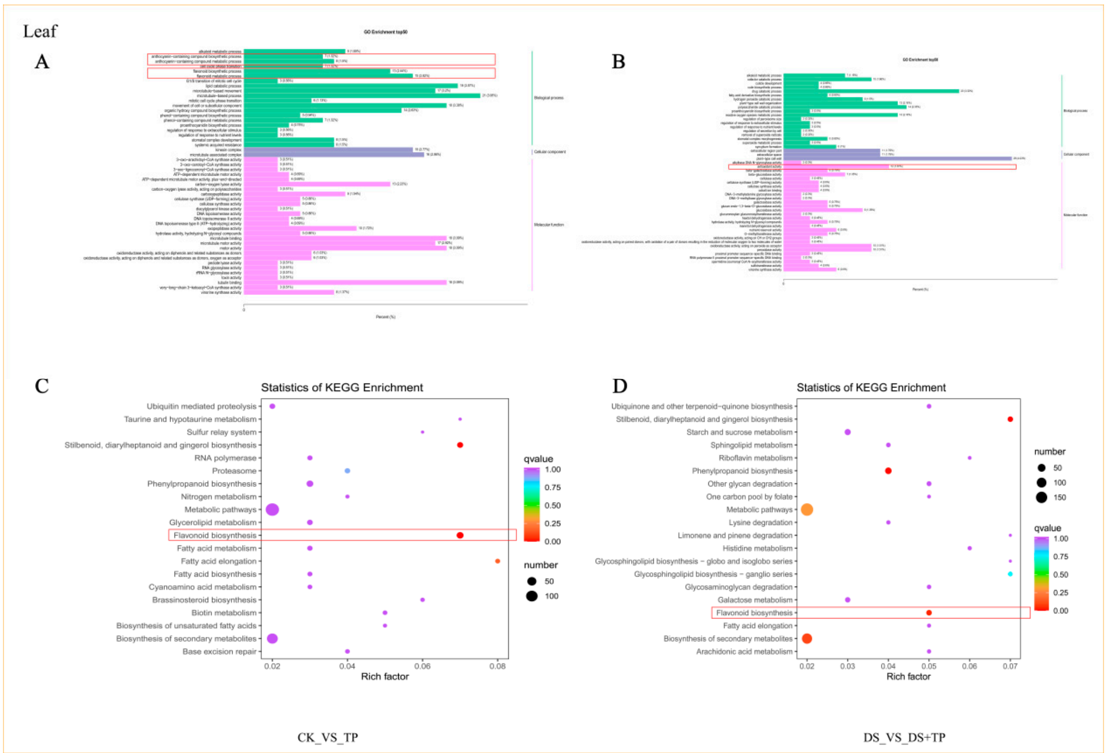

Figure S2. Plot of GO and KEGG enrichment analysis in leaves of different treatments. Leaf (**A**) CK_VS_TP and (**B**) DS_VS_DS+TP GO enrichment analyses top50 histograms; leaf (**C**) CK_VS_TP and (**D**) DS_VS_DS+TP KEGG enrichment analyses scatter plots. Red boxes indicate important concerns. Green for biological processes; purple for cellular components; pink for molecular functions. The ordinate represents the KEGG pathway. The abscissa represents the Rich factor. Rich factor refers to the ratio of the enriched differential genes (Sample number) to the annotated genes (Background number) in the pathway. The greater the Rich factor, the greater the degree of enrichment. The larger the dot, the higher the number of differential genes enriched by the pathway. P-value: p-value for significance test; Corrected_P-value (qvalue): p-value after correction for multiple hypothesis test The smaller the qvalue, the redder the color of the point, indicating the more significant the enrichment. CK: control group, normal watering; TP: exogenous tea polyphenol pretreatment and normal watering; TP+DS: exogenous tea polyphenol pretreatment and drought treatment; DS: drought treatment.

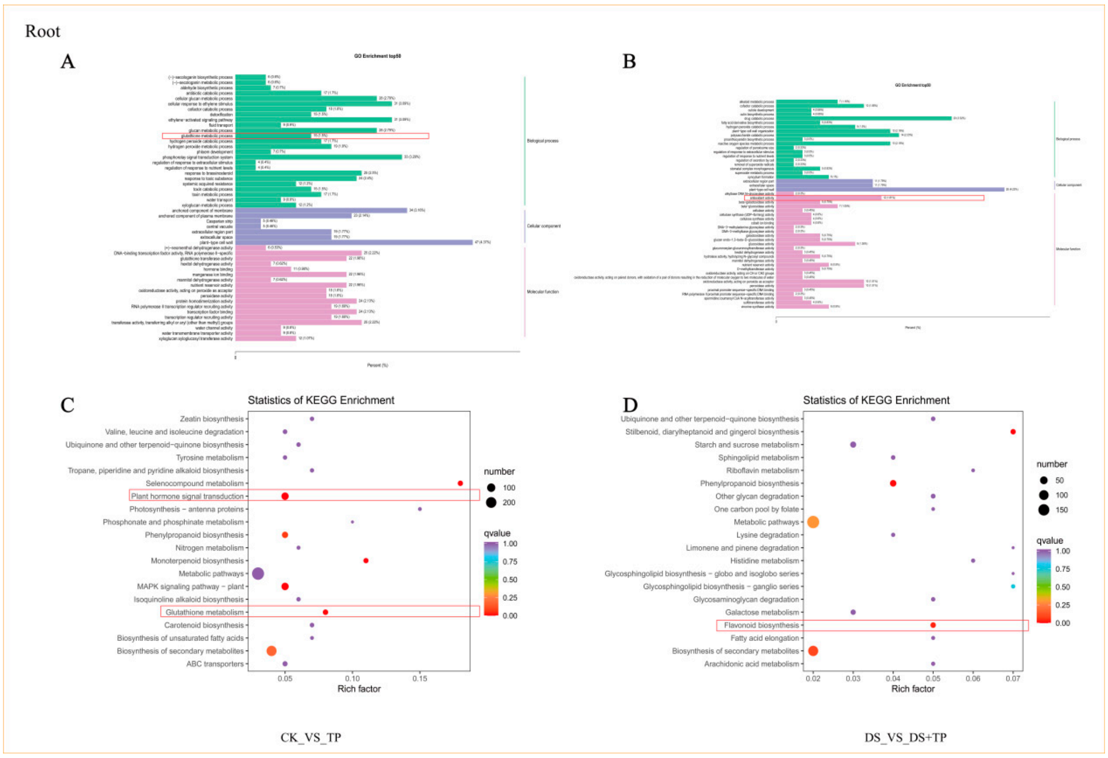

Figure S3. Plot of GO and KEGG enrichment analysis in roots of different treatments. Root (**A**) CK_VS_TP and (**B**) DS_VS_DS+TP GO enrichment analyses top50 histograms; root (**C**) CK_VS_TP and (**D**) DS_VS_DS+TP KEGG enrichment analyses scatter plots. Red boxes indicate important concerns. Green for biological processes; purple for cellular components; pink for molecular functions. The ordinate represents the KEGG pathway. The abscissa represents the Rich factor. Rich factor refers to the ratio of the enriched differential genes (Sample number) to the annotated genes (Background number) in the pathway. The greater the Rich factor, the greater the degree of enrichment. The larger the dot, the higher the number of differential genes enriched by the pathway. P-value: p-value for significance test; Corrected_P-value (qvalue): p-value after correction for multiple hypothesis test The smaller the qvalue, the redder the color of the point, indicating the more significant the enrichment. CK: control group, normal watering; TP: exogenous tea polyphenol pretreatment and normal watering; TP+DS: exogenous tea polyphenol pretreatment and drought treatment; DS: drought treatment.

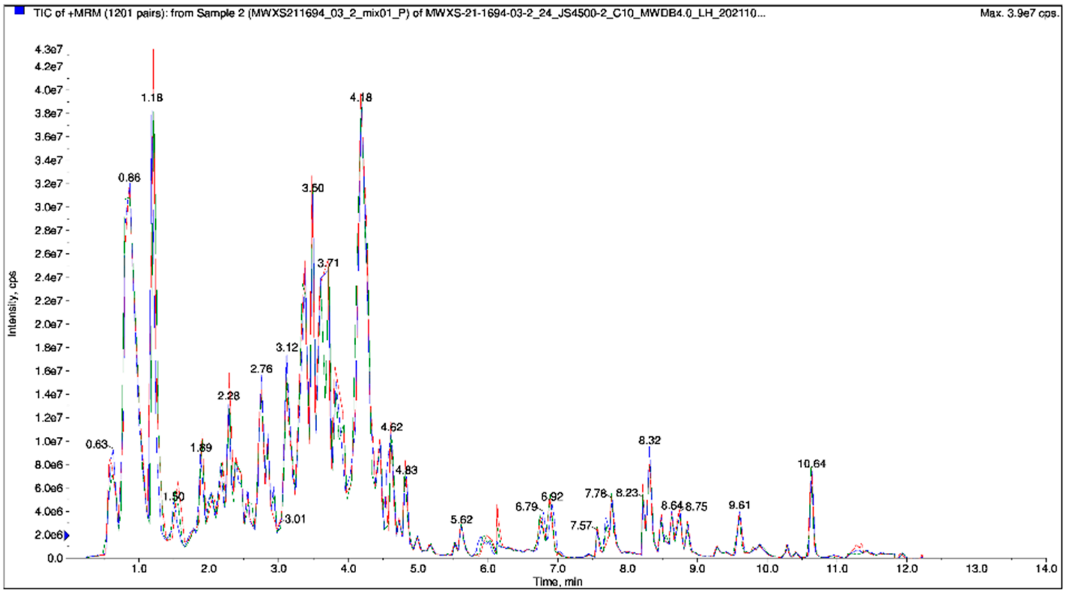

Figure S4. QC sample mass spectrometry detection TIC overlay. In the process of analysing the samples of tea tree roots and leaves, one QC sample was inserted in every 10 samples analysed for detection, and the total ion flow plots analysed by mass spectrometry detection of the QC samples overlapped well, demonstrating the high stability of the instrument and the reliability of the data results.

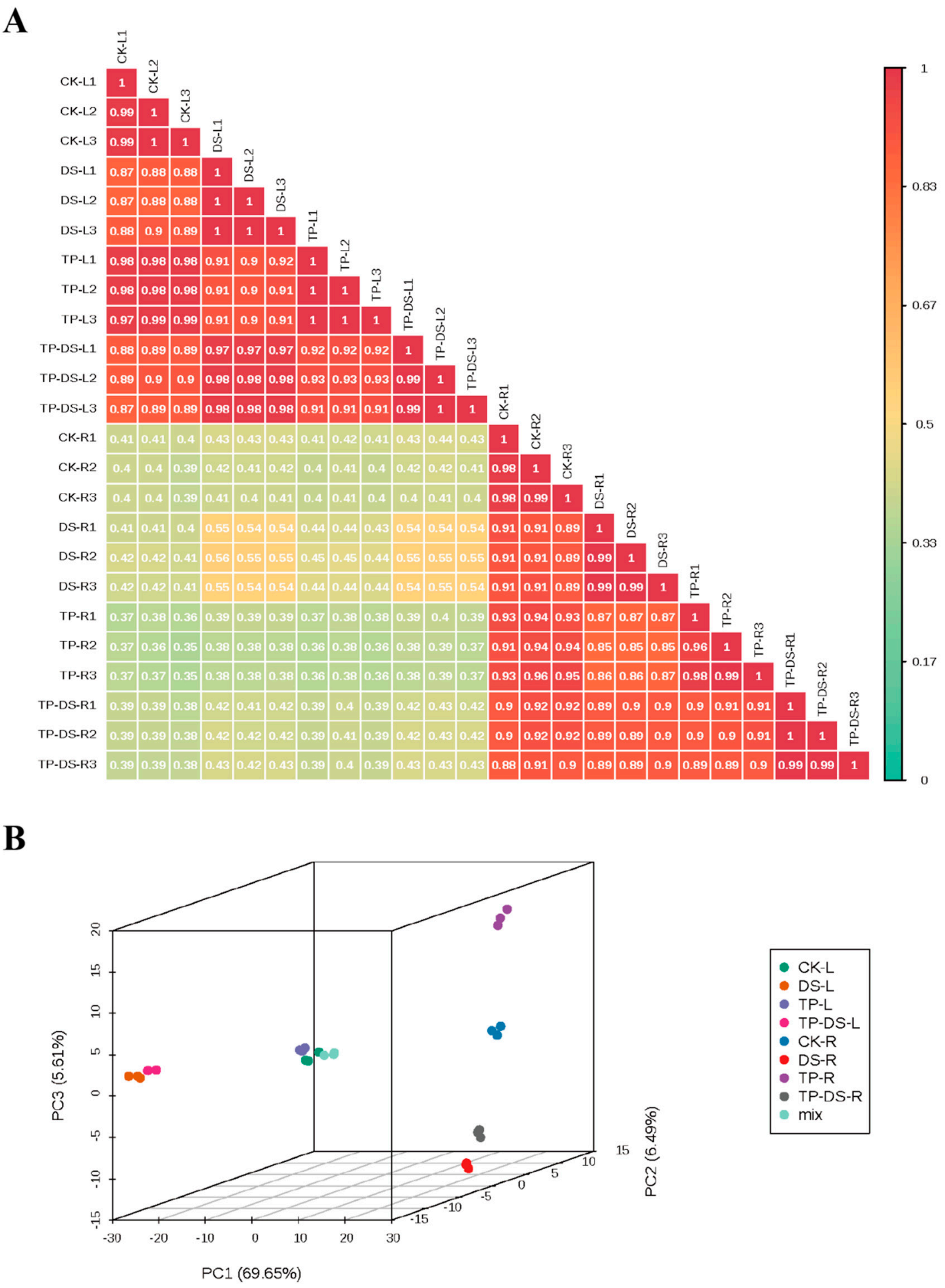

Figure S5. Metabolome correlation analysis of leaves and roots under different treatments. (**A**) Pearson correlation analysis of metabolomic data, (**B**) principal component analysis. CK: control group, normal watering; TP: exogenous tea polyphenol pretreatment and normal watering; TP+DS: exogenous tea polyphenol pretreatment and drought treatment; DS: drought treatment; L: leaves; R: roots.

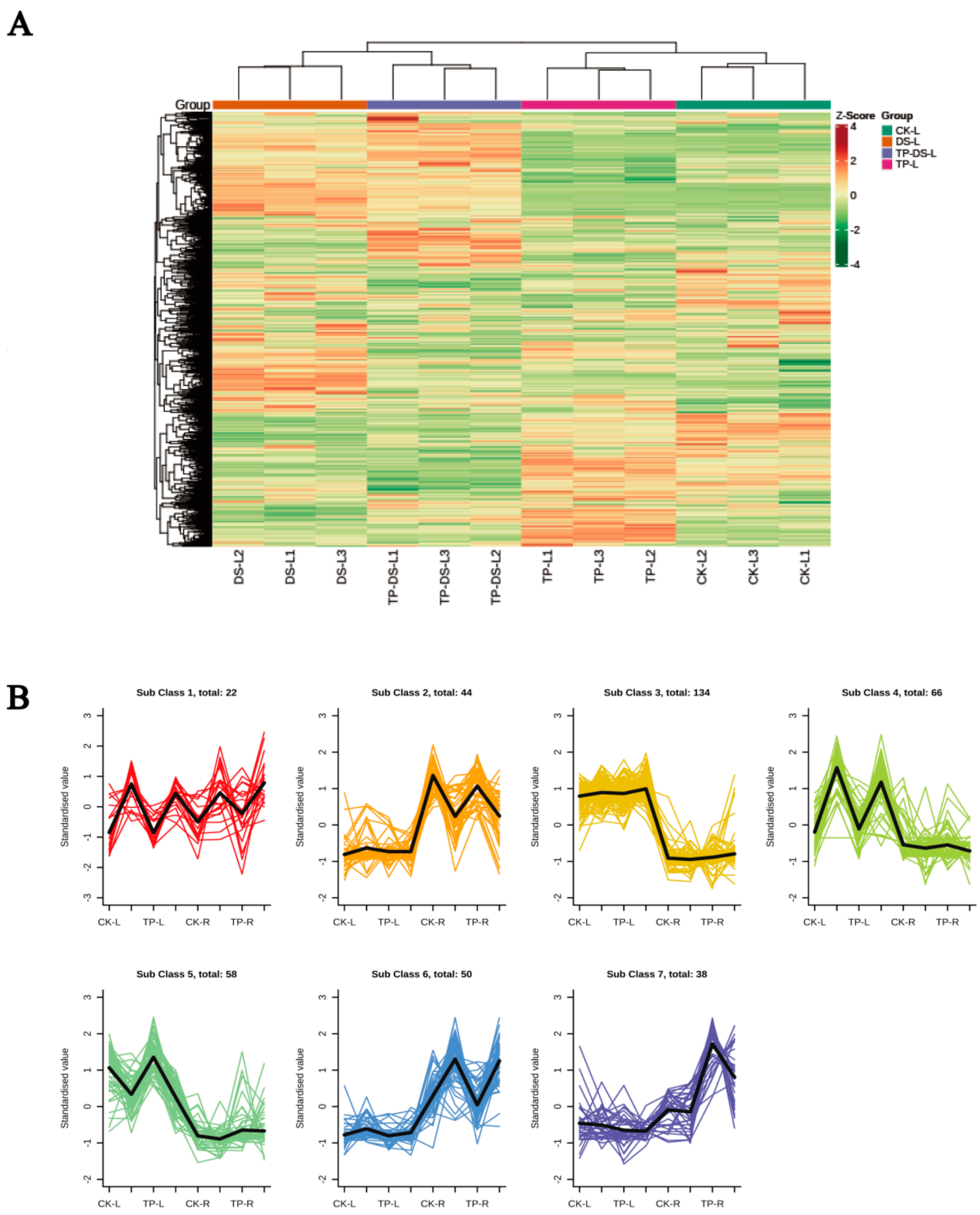

Figure S6. Metabolomic analysis of leaves and roots under different treatments. (**A**) Heat map of leaf metabolome clustering and (**B**) K-MEAN analysis of 1055 metabolites. CK: control group, normal watering; TP: exogenous tea polyphenol pretreatment and normal watering; TP+DS: exogenous tea polyphenol pretreatment and drought treatment; DS: drought treatment; L: leaves; R: roots.

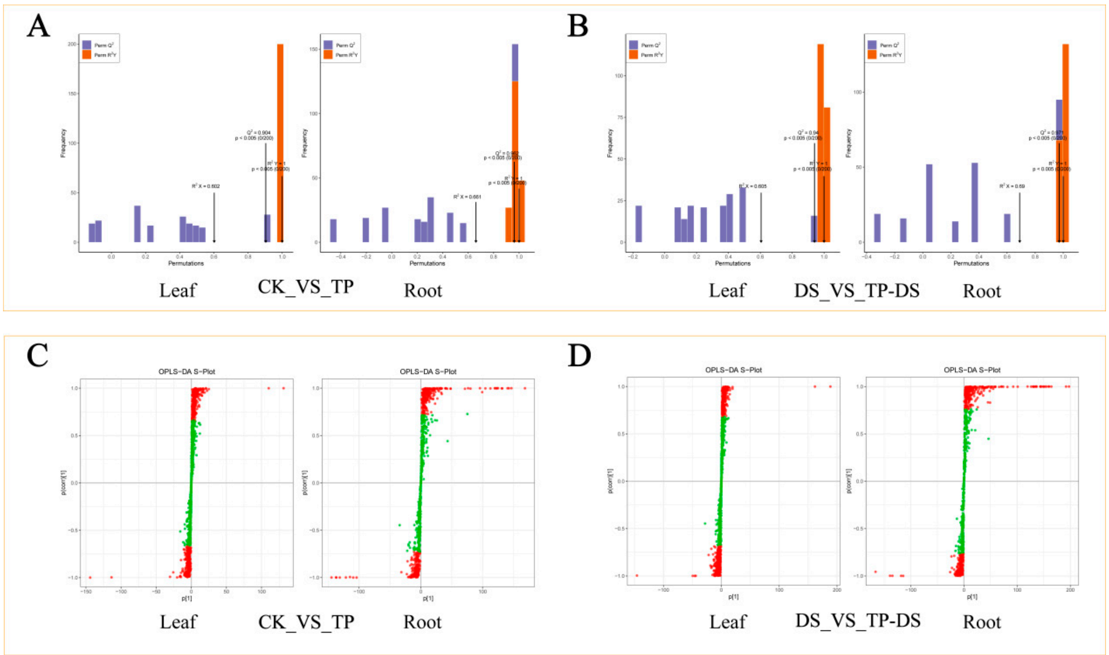

Figure S7. Evaluation of metabolomic models for leaves and roots under different treatments. The projected (**A**,**B**) importance (VIP) values of ectopic silica in each comparison group were obtained by (**C**,**D**) OPLS-DA. The predictive parameters of the evaluated models are R2X, R2Y and Q2, which were validated for each set of models, and the minimum value of Q2 was 0.904 model excellent for further analysis. CK: control group, normal watering; TP: exogenous tea polyphenol pretreatment and normal watering; TP+DS: exogenous tea polyphenol pretreatment and drought treatment; DS: drought treatment; L: leaves; R: roots.

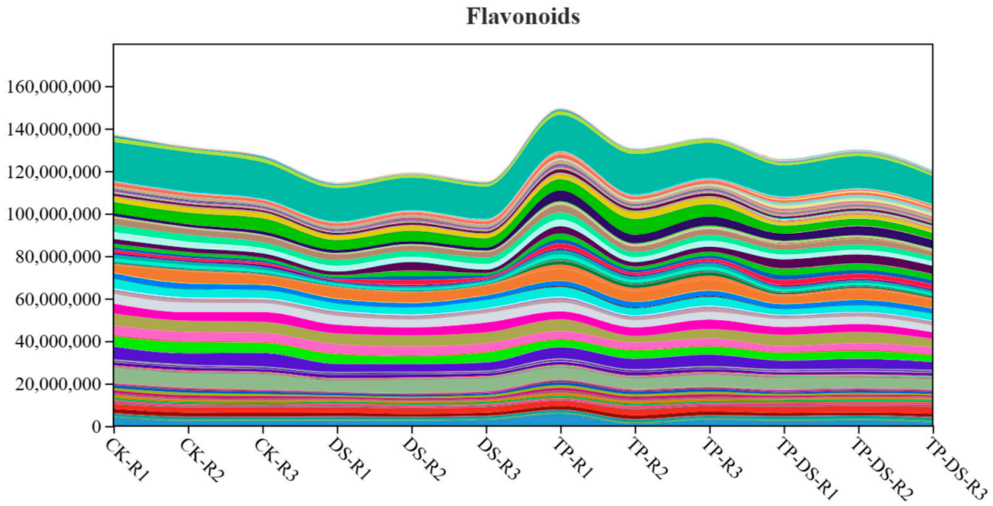

Figure S8. Rivers plot of flavonoid compound abundance in roots. With each color representing a compound. CK: control group, normal watering; TP: exogenous tea polyphenol pretreatment and normal watering; TP+DS: exogenous tea polyphenol pretreatment and drought treatment; DS: drought treatment; R: roots.

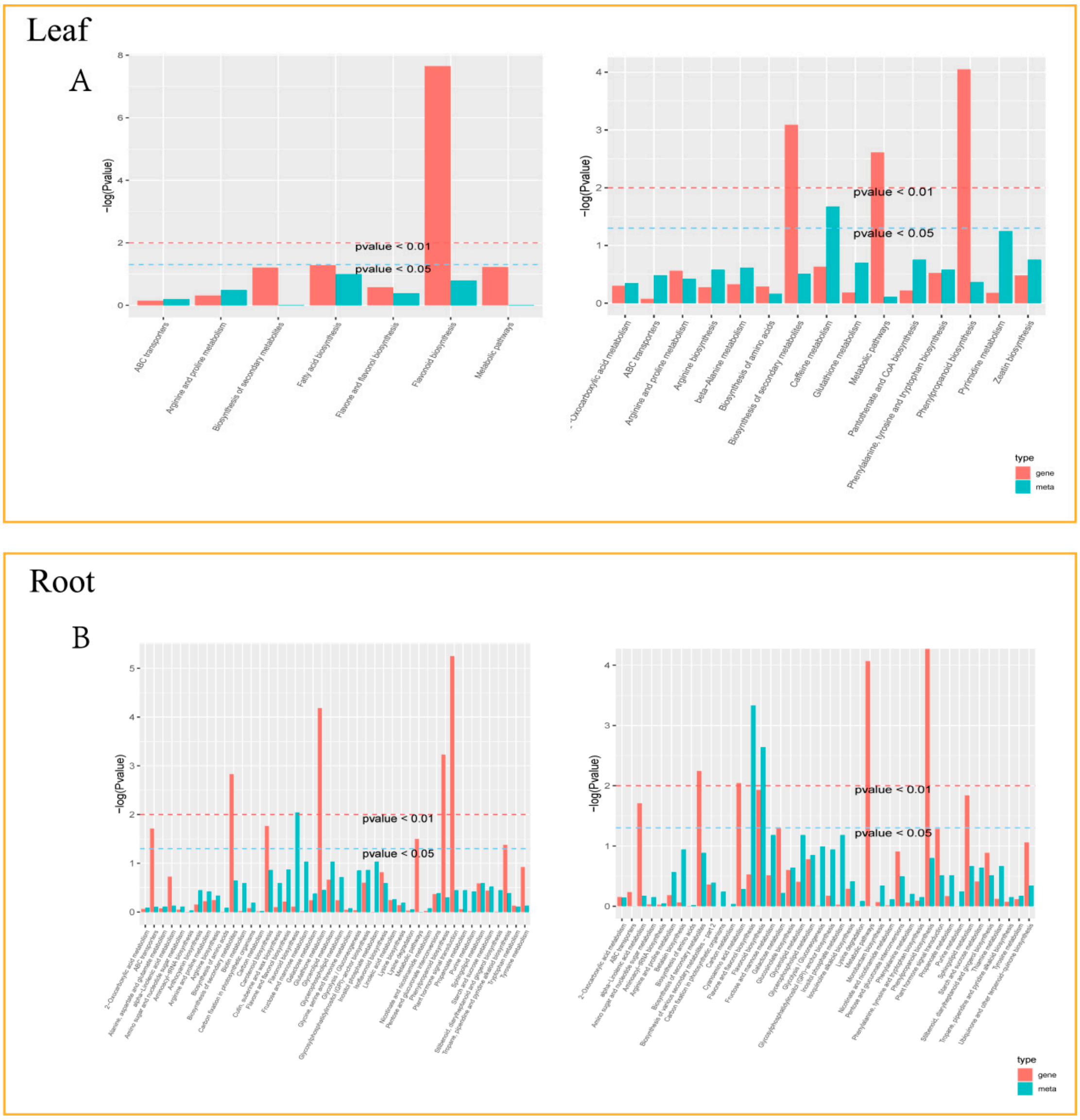

Figure S9. Joint analysis of transcriptional metabolism in leaves and roots under different treatments. Differential genes and differential metabolites were jointly analysed for the first 20 KEGG-enriched pathway histograms in leaves (**A**) and roots (**B**). The left panel shows CK_VS_TP, and the right panel shows DS_VS_DS+TP. CK: control group, normal watering; TP: exogenous tea polyphenol pretreatment and normal watering; TP+DS: exogenous tea polyphenol pretreatment and drought treatment; DS: drought treatment; L: leaves; R: roots.
